# Considering Metabolic Context in Enzyme Evolution and Design

**DOI:** 10.1021/acs.biochem.5c00165

**Published:** 2025-08-05

**Authors:** Dhiraj Dokwal, Philip M. Brown, Karolina Filipowska, Kimberly A. Reynolds

**Affiliations:** † Green Center for Systems Biology—Lyda Hill Department of Bioinformatics, 12334 The University of Texas Southwestern Medical Center, Dallas, Texas 75230, United States; ‡ Department of Biophysics, The University of Texas Southwestern Medical Center, Dallas, Texas 75230, United States

**Keywords:** Escherichia coli, dihydrofolate reductase, NADPH, CRISPR, deep mutational scanning

## Abstract

Enzymes are often treated as isolated molecular entities when measuring their biochemical activity and designing synthetic sequences. Yet inside the cell, enzymes interact through shared metabolite pools, physical binding, and regulatory feedback. This coordination is necessary for responsive, cohesive metabolic behavior. Moreover, these interactions place constraints on enzyme activity, specificity, abundance, and ultimately sequence. Defining these interaction-mediated constraints is important to understand metabolic evolution and to design synthetic systems. For example, the influence of the cellular and environmental milieu becomes evident when a heterologous enzyme from one species fails to function properly upon transfer to a new host. In this review we consider how cell context shapes enzyme evolution, and in turn, how variations in enzyme biochemistry, biophysics, and abundance impact cell fitness.

## Graphical abstract

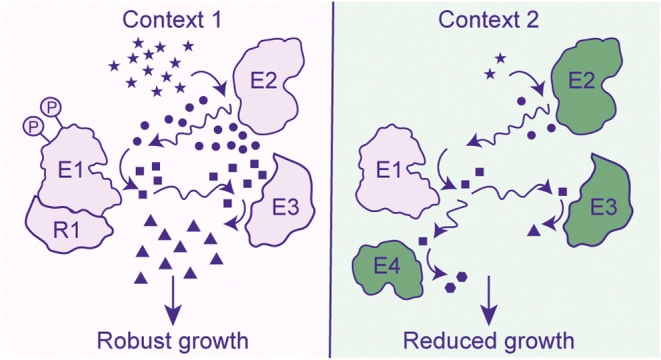

## Introduction

The combined activity of hundreds, even thousands, of enzymes gives rise to metabolism—the collection of chemical processes that transform exogenous nutrients into cellular components and energy. Historically, *in vitro* biochemical characterization of component enzymes has played a central role in understanding metabolic mechanism. Individual enzymes can be expressed, purified, and characterized in exquisite detail, painting a multidimensional portrait of their activity and function. For example, the complete mechanism of *Escherichia coli* dihydrofolate reductase (DHFR) comprises 5 catalytic intermediates and 27 distinct rate constants, including the *k*_on_ and *k*_off_ of both substrate and cofactor.^1^ In many cases, enzymes are quantitatively described in terms of biochemical steady-state parameters (*k*_cat_ and *K*_m_), biophysical measurements of stability (*T*_m_, ΔΔ*G*_fold_), ligand binding affinities (*K*_d_), inhibition constants (*K*_i_), and more.^2,3^ Over the last several decades, catalytic parameters have been measured for over 8000 enzymes under a diversity of experimental conditions.^4^ This work has yielded fundamental insights into the biophysical origins of catalysis and begun to reveal the connection between sequence variation and catalytic activity. Moreover, recent advances in microfluidics have enabled high-throughput measurement of biochemical parameters across thousands of sequence variants.^5^ In parallel, there has been rapid progress in extracting quantitative biophysical constants and enzyme activity information from high-throughput growth based assays.^6–9^ Together these developments suggest that we are on the cusp of a new era replete with quantitative biochemical and biophysical data.

Yet it remains deeply unclear how variation in these biochemical and biophysical parameters shape metabolic function, phenotype, and ultimately organismal fitness. Because enzymes function in the larger context of the cell, interactions among proteins have the potential to buffer or amplify the impact of biochemical variation on complex phenotypes like growth rate or metabolic yield.^10–12^ Consequently, the relationship between enzyme activity, intracellular abundance, and phenotype is nonlinear and dependent on genetic background.^13,14^ For most enzymes it is unclear what values of protein abundance, thermal stability (Δ*G*_fold_), and catalytic activity (*k*_cat_, *K*_m_) suffice to sustain metabolic pathway flux and support cell growth. We often lack even orders-of-magnitude level bounds on these fundamental biochemical parameters—we do not have a sense of which enzyme properties must be precisely tuned and which are robust to variation. Nevertheless, we expect some balance between robustness and adaptability in the space of biochemical parameters to ensure a plastic, evolvable system.^15–17^ Indeed, theoretical work indicates that biological systems can be well-described by so-called “sloppy models”: models wherein a few stiff parameters matter enormously to system behavior, while many other values are very loosely constrained.^18^ Mapping the constraints on enzyme biochemistry and biophysics is a key first step toward our foundational understanding of metabolic evolution. It is also necessary to achieve practical goals like the rational design of synthetic cellular systems and interpretation of disease-associated mutations.^19,20^

With this inspiration, we discuss recent work concerning the impact of cell context on the evolution of enzyme catalysis, abundance, and sequence. Likewise, we consider the converse—how variation in sequence, catalysis and abundance impacts cell function and organismal fitness. We emphasize the role of intermolecular interactions in shaping metabolic and enzyme evolution and distinguish among two broad interaction types: physical binding and biochemical coupling (Figure 1A,B). Physical binding is the more familiar case of direct protein–protein interaction through the formation of an interface (Figure 1A). In metabolism, relatively high-affinity physical interactions (characterized by nano- or micromolar binding constants) play an important role in regulation,^21,22^ colocalization,^23–25^ and phenomena like substrate channeling.^26^ A growing body of evidence also suggests potential for lower-affinity transient interactions or agglomerates as an organizing force.^27–30^ In contrast, biochemical interactions are not mediated by direct binding. Instead, these emerge via coupling through a shared metabolite pool–this could be a substrate, a product, or both (Figure 1B). The need to preserve flux through a pathway while avoiding the accumulation of toxic intermediates or depletion of key molecules can constrain the relative activity of enzymes, and potentially drive coevolution across multiple genes.^31,32^ These physical and biochemical interaction types are of course not mutually exclusive, and both serve to constrain available evolutionary paths. By considering the role of cell context in shaping enzymatic function, we hope to better understand the evolution of metabolism and improve the efficiency of designing synthetic sequences and cell systems.

**Figure 1:**
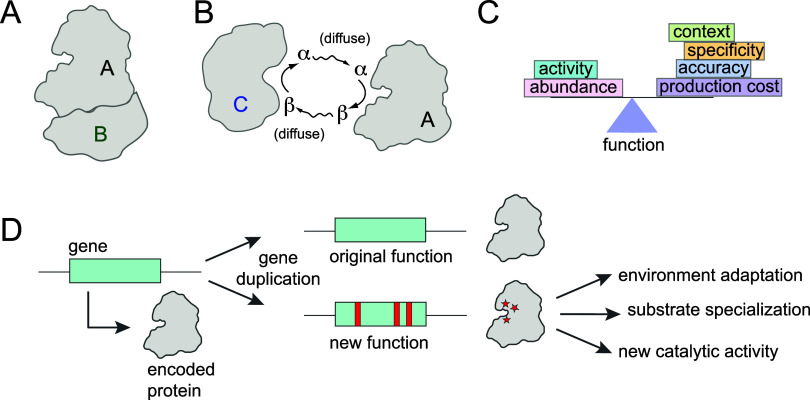
Evolution of new enzymatic activity and associated functional constraints. (A) Physical interactions between proteins are mediated through a binding interface. This constrains the sequence of amino acid positions directly at the interface, as well as distal positions that indirectly modulate binding affinity and specificity. (B) Biochemical interactions are mediated through a shared metabolite pool. This constrains the relative activity and abundance of the two enzymes and can potentially shape sequence variation at amino acid positions coupled to intracellular abundance and catalytic activity. (C) Enzyme function must balance optimizing the activity and abundance of the enzyme against constraints on specificity, accuracy, and production cost. Cellular context (including factors like chaperone/protease availability) can additionally modulate this relationship. (D) Gene duplication and divergence facilitates the evolution of new substrate specificity, environment-specific adaptations, and new catalytic function.

### Environmental and Metabolic Context Shapes Enzyme Biochemical Parameters

To highlight the influence of cell context on enzyme function, consider xenolog gene swaps, where a single gene is replaced by its homologue from another organism. Kachroo and colleagues found that 53% of 414 essential yeast genes could not be replaced by their homologous human counterparts.^33^ Likewise, only 38% of over 1200 natural chorismate mutase sequences were able to complement growth in a chorismate mutase deficient *E. coli* strain.^34^ In both of these cases, homologous genes are presumably enzymatically active and functional in their native context yet show limitations in transfer to a new organism. This limited fungibility emerges from variation in metabolic context and the environmental demands placed on a particular organism.

Environmental changes, such as fluctuations in temperature,^35,36^ availability of oxygen,^37^ anthropogenic toxins,^38–40^ or the presence of antibiotics,^41–43^ continually drive the emergence of new biochemical variation and shape the constraints on enzyme activity. During adaptation, enzyme properties are subject to trade-offs in specificity, stability, flexibility, and activity (Figure 1C). Perhaps the most classic example of environmentally driven changes in enzyme biophysics is thermal adaptation. Thermal adaptation is sometimes associated with an activity-flexibility trade-off, in which cold adapted enzymes retain activity at lower temperatures through enhanced flexibility.^44^ Depending on the enzyme family, this tuning can be accomplished by limited sequence variation in the active site, or via distributed, remote surface mutations that allosterically tune activity. For example, work from Pinney et al. found that mutational variation at a single active site residue in ketosteroid isomerase provided either a stronger hydrogen bond donor (and higher activity) for cold-inhabiting variants, or a weaker hydrogen bond donor with improved folding for warm-adapted variants.^35^ In contrast, variation at structurally remote (and often solvent exposed) sites has been reported to tune flexibility and potentially thermal stability in adenylate kinase,^36^ lactate dehydrogenase,^44^ and trypsin.^45^ Statistical analysis of sequence variation across homologues alongside metadata on preferred organism growth temperature has provided one route to identify the sequence changes associated with stability and/or temperature adaptation.^46,47^

Beyond thermal adaptation, enzyme function is also subject to trade-offs in efficiency and specificity. It is thought that early enzymes exhibited broad substrate specificity that was narrowed over time due to negative selection against off-target reactions.^48^ However, increased specificity often comes at a cost of decreased processivity or efficiency.^49^ Indeed, diffusion-limited reaction rates and perfect accuracy are not common; most enzymes exhibit “moderately efficient” values of *k*_cat_ (∼10 s^–1^) and *K*_m_ (∼100 μM).^50^ Measurements of absolute metabolite abundances in *E. coli* indicate that, for many enzymes, *K*_m_ values lie below physiological substrate concentrations, suggesting that active sites are frequently saturated and that enzymes often operate near their maximal turnover rates.^51^ This trend may reflect a selective upper bound on substrate affinity. However, it is important to note that a substantial fraction of reactions are substrate-limited, particularly in core pathways like glycolysis, where substrate concentrations are often closer to the *K*_m_. In these cases, changes in metabolite concentrations can significantly impact reaction rates and the direction of metabolic flux. Moreover, even when enzymes’ turnover rates are largely saturated, variations in metabolite concentrations can influence fluxes by competitive inhibition, as structurally similar metabolites often share binding sites.^52^

The constraints on activity and specificity vary with metabolic context. Meta-analysis of *in vitro* biochemical data^50^ and metabolic flux analysis^52^ found that essential enzymes engaged in highly connected core metabolic processes were statistically enriched to be both more specific and catalytically proficient. In contrast, enzymes in secondary metabolism were relatively less catalytically proficient and showed higher promiscuity. Further biochemical characterization of orthologous genes sampled from diverse species is needed to help define the evolutionary constraints on thermal stability, *k*_cat_, and *K*_m_ across distinct cellular processes and organismal lifestyles/environmental niches.

### Physical and Biochemical Interactions Alter Enzyme Mutational Landscapes

Given that cell context constrains enzyme biochemistry, it must also shape protein sequence. Yet it remains an open question to understand how sequence encodes variation in specific biochemical or biophysical properties. Both direct physical interactions between enzymes and regulators (Figure 1A), or biochemical interaction between enzymes sharing metabolite pools (Figure 1B) can restrict the space of viable sequences, and may give rise to distinct signatures in sequence variation. Statistical analysis of protein multiple sequence alignments provides one route to begin mapping the relationship between amino acid conservation, coevolution, and particular functional or phylogenetic clades.^53,54^ The basic principle of this approach is that evolutionary conservation reports on functional importance, while coevolution reflects potential interactions among amino acids. Using dimension reduction approaches from linear algebra (e.g., singular value decomposition, or SVD), coevolution among particular groups of amino acids can be associated with particular functional divergences. For example, coevolutionary analysis of the S1A serine proteases identified distinct residue groups (or “sectors”) associated with catalytic specificity, catalytic activity, and thermal stability.^47^ This work suggests the possibility that some enzymes have a modular design, allowing for orthogonal tuning of different biochemical properties with mutations in distinct structural regions. Interestingly, high-throughput biochemical measurements for the enzyme PafA also revealed distinct structural regions that tuned specific aspects of function.^5^

Experimental techniques like deep mutational scanning (DMS), directed evolution, and biophysical characterization can also serve to map the connection between sequence, cell context, and function. To illustrate, we highlight Dihydrofolate reductase (DHFR) as one emerging model system. DHFR is an essential enzyme in folate metabolism that catalyzes NADPH-dependent reduction of dihydrofolate to tetrahydrofolate.^55^ Because this enzyme plays a central role in cell growth, DHFR is a common target of antibiotics (e.g., trimethoprim) and chemotherapeutics (e.g., methotrexate). Moreover, DHFR function is readily assessed through growth-based assays; initial work found a quantitative relationship between enzyme biophysical parameters, DHFR function, and trimethoprim resistance.^56^ Other studies have expanded to characterize sequence-fitness landscapes with DMS. In these experiments, a cell-based functional assay of enzyme activity is coupled to next generation sequencing to measure the effect of thousands of mutations on cell fitness (growth rate).^57^ Mutations with deleterious effects on growth rate are presumed to be deleterious to enzyme function or folding. For example, DMS of *E. coli* DHFR (*ec*DHFR) identified a class of mutations whose effects depend on interactions with *Lon* protease, a quality control enzyme.^58^ DHFR is a client of Lon^59–61^ and Lon is found in some (but not all) strains of *E. coli*. In the absence of Lon, a handful of DHFR mutations were beneficial to cell fitness; several of these were experimentally shown to improve catalytic activity. However, in the presence Lon, many of these mutations were associated with increased Lon degradation, lowered DHFR stability, and *E. coli* reduced fitness. Thus, interactions with the proteostasis machinery impact DHFR activity and sequence. In addition to the direct physical interaction with Lon, the impact of DHFR mutations on fitness is shaped by biochemical interactions with the upstream enzyme Thymidylate Synthase (TYMS)^31,62,63^ (Figure 1B). Here, changes in one enzyme’s activity modulated the selective pressures on the other. Selection experiments in *E. coli* found that TYMS activity cannot strongly exceed that of DHFR, due to a requirement to preserve shared metabolite pools. This interaction modulated the available routes of antibiotic resistance: inhibition of DHFR with trimethoprim can be partly compensated by loss of function mutations in TYMS.^31,64^ Other work has suggested that DHFR interacts with other folate metabolic enzymes to form metabolons.^65^ Finally, eukaryotic and prokaryotic orthologs of DHFR were shown to have distinct catalytic mechanisms that may originate from cellular changes in cofactor availability.^66^ While *ec*DHFR undergoes a five step catalytic cycle that involves the exchange of spent cofactor (NADP+ for NADPH) before binding fresh susbtrate, the human enzyme (*hs*DHFR) is able to reload substrate before exchanging the cofactor. Interestingly, the bacterial cell environment is rich in NADP^+^, while NADPH is the more abundant species in eukaryotes. NADPH-NADP^+^ exchange is thus less efficient in bacteria, and mechanisms to avoid futile catalytic cycles without NADPH must exist. Interestingly, *ec* and *hs*DHFR share a very similar structure, and changes in catalysis are encoded in their nuanced dynamic movements.^67,68^ Altogether, these results indicate that DHFR function is modulated by several aspects of cell background, including Lon protease availability, TYMS activity, and cofactor concentrations. Given this, it is perhaps not surprising that in a study of 35 DHFR variants sampled from mesophilic bacteria, 31 exhibited a fitness defect when transferred into *E. coli* lacking an endogenous DHFR.^65^ Likewise, hsDHFR does not complement *E. coli* growth under strong selective conditions.^66^ In many cases, the associated fitness defects can be overcome by laboratory evolution and a small number of sequence changes. Nevertheless, this interplay between cell background and enzyme function has implications for both restricting natural horizontal gene transfer (HGT) and functional screening of synthetic (designed) sequences.

### Gene Duplication and Divergence are Facilitators of Evolutionary Innovation

The emergence of new enzymatic activities can often be traced to gene duplication and divergence^69^ (Figure 1D). As this process creates redundancy in the genetic material, one copy of the gene can maintain the original function, freeing the other from evolutionary constraints and allowing for the accumulation of mutations. These sequences can give rise to proteins serving new functions. Over time, duplicated genes can undergo amplicon remodeling, where the two gene copies move to distinct genomic regions, facilitating further evolutionary changes.^70^ As these changes happen within the broader context of metabolic and regulatory networks, gene duplication can impose a burden on the host organism. Thus, the newly duplicated gene may not immediately contribute to fitness, or even cause a fitness defect.^71^ As a result, the organism may need to evolve further to optimize gene function and reduce any negative impacts, balancing the benefits of the enzyme’s activity with the fitness cost of its expression.

### Constraints on Absolute Enzyme Abundance

The connection between enzyme abundance and fitness (cell growth rate) is often considered as a cost-benefit trade-off.^72^ (Figure 2A). Creating metabolic enzymes benefits the cell by increasing flux through key reactions but also costs energy and resources. The *expression-benefit* relationship is often governed by a hyperbolic function. At low enzyme concentrations, small increases in enzyme concentration confer a fitness benefit, while at high concentrations the expression-dependent fitness effect plateaus. In contrast, the *expression-cost* relationship is expected to scale near-linearly with the increase in enzyme concentrations^73^ (Figure 2A). For example, the ATP used to transcribe mRNA, assemble amino acids, and fold protein (via chaperones) should be roughly proportional to the amount of protein produced. Overexpression of a nontoxic exogenous protein in yeast showed evidence of nitrogen starvation due to protein overproduction.^74^ Indeed, transcription or translation can limit protein production and induce a fitness cost depending on whether cells are grown in phosphate- or nitrogen-limited media.^75^ Finally, overexpression can drive gene-specific toxicity.^65,71–73,76–78^ Toxicity can be attributed to a number of mechanisms including mass-action-driven interaction promiscuity,^76^ improper protein complex stoichiometry,^76,77,79^ accumulation of toxic intermediate metabolites,^20,31,80,81^ and more. When cost, benefit, and toxicity combine they can give rise to a variety of abundance-fitness relationships. These can be mathematically described as monotonic sigmoidal functions,^82,83^ but can also take the form of nonmonotonic curves with an optimum expression value described by impulse functions.^84^ This phenomenon is sometimes referred to as hormesis, and seems more prevalent under stressful conditions^85^ (Figure 2A).

**Figure 2:**
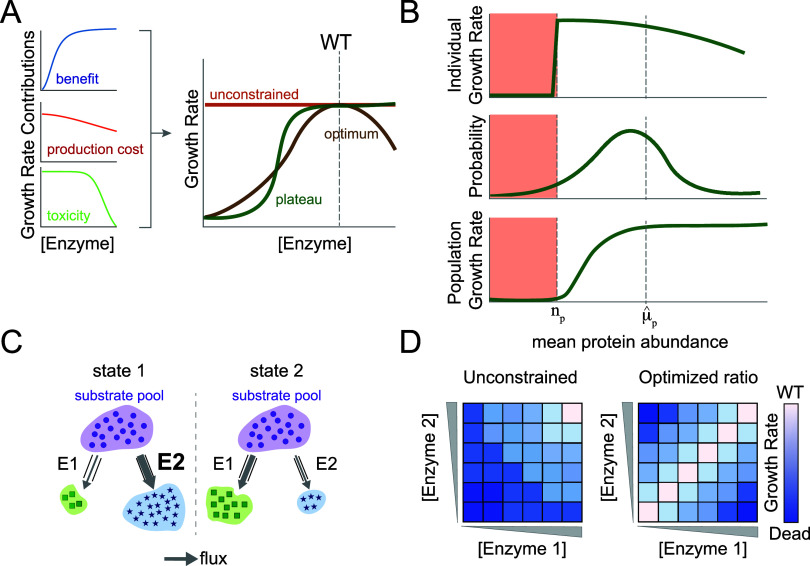
Connection between enzyme abundance and growth rate. (A) The metabolic benefit of increased enzyme expression (blue curve) can be offset by protein production cost (red curve) and gene-specific toxicities (green curve). These factors combine to shape the overall relationship between enzyme abundance and growth rate (right panel). (B) Transcriptional noise is proposed to shape the mean expression level of proteins (μ̂_p_). Individual cells have a threshold protein abundance necessary for viability (*n*_p_) (green curve, top panel). Stochasticity in transcription yields cell-to-cell variation in the transcript numbers, such that a population of cells samples a probability distribution of intracellular protein abundance (green curve, middle panel). Proteins should thus be expressed well above the threshold *n*_p_ to avoid incurring a fitness cost (green curve, bottom panel). In the bottom plot, the population growth rate is a function of mean protein abundance, where the population growth rate is a convolution between the single-cell growth rate at each level of protein abundance (top panel) and the population distribution of protein abundance (middle plot). Figure adapted from Lo et al.^86^ (C) Biochemical interactions across metabolism can create nonintuitive dependencies in relative abundance. In this example, a shared substrate pool (purple circles) couples the activity of two enzymes E1 and E2 (represented by gray schematized “pipes”, pipe width is used to indicate enzyme intracellular abundance). Flux through E2 (gray arrow) is higher under conditions of equivalent expression (state 1), given that E2 has a higher *k*_cat_ or lower *K*_m_ than E1. In state 1 of this model, we see that E2 is substrate-saturated such that the flux of the reaction catalyzed by E2 is limited by enzyme abundance, whereas the reaction catalyzed by E1 is not. In this case, a decrease in the abundance of E2 (state 2) limits the flux through this reaction and simultaneously increases the accumulation of the substrate. This limitation in E2 allows E1 to reach saturation and consequently increases the flux through E1′s reaction. (D) Biochemical interactions can impose constraints on relative enzyme abundance. Each heat map shows growth rate (blue-white coloring) as a function of abundance in E1 and E2. In some cases, the relative abundance of enzymes is seemingly unconstrained (left panel) with only a constraint on the absolute abundance of individual enzymes. In other instances, stoichiometric constraint on enzyme abundance creates an optimal expression ratio (right panel).

Characterizing the relationship between enzyme abundance, metabolic flux, and growth rate requires an experimental mechanism to tune abundance and measure the impact on growth. Some of the earliest efforts made use of Neurospora heterokaryons, which allowed one to manipulate the fraction of active enzyme by varying the nuclear ratio of WT and loss-of-function enzyme alleles but did not allow more direct control on expression.^10^ This was followed by work inserting an inducible promoter proximal to a gene-of-interest^87,88^ and characterizing fitness as a function of gene expression,^89^ a strategy later expanded to create large collections of strains with gradated expression levels.^84^ Moriya et al. developed the “genetic tug-of-war” method in yeast where the copy number of a gene and its native promoter and terminator are increased in yeast strains until a detrimental fitness effect was observed,^90^ and Moriya extended these studies to define the permissible range of expression for the *Saccharomyces cerevisiae* genome.^77^ Recent efforts use next generation sequencing with CRISPR-interference (CRISPRi) to map the impact of gene expression changes in high throughput.^82,83,91–94^ Gene-specific repression is achieved by targeting a catalytically dead Cas-9 protein (dCas9) to a specific gene with a single guide RNA (sgRNA). The sgRNA has 20 base pairs of homology to the target gene. The knockdown effect of dCas9 is titrated through introduction of mismatches in the homology region of the sgRNA; the mismatches reduce dCas9 complex affinity for the target gene and increase transcription relative to a guide RNA with full homology. Large scale CRISPRi screens with mismatch or titrating sgRNAs found that the relationship between gene expression and growth rate is highly gene specific, varying with gene function(s), environmental conditions, and the organism’s evolutionary context.^84,85,92–94^ A comparison of knockdown effects across two bacterial species indicated that the shape of these relationships are preserved for genes involved in similar cellular processes.^82^ For metabolic enzymes native protein levels are commonly expressed in excess of their limiting abundance, consistent with classic models of metabolic pathways only having one limiting reaction at a time^87^ or maintaining robustness given stochasticity of expression.^86^ Generally, these studies contribute to our understanding of the quantitative properties of gene essentiality, revealing targetable vulnerabilities in microbial genomes^95^ and building quantitative models of expression-fitness landscapes.^14^

### The Impact of Stochastic Transcription on Expression Level

The cost-benefit function described above focuses on the intrinsic functional benefit and production cost of a protein. However, other aspects of the central dogma—including the rates of transcription, translation, mRNA decay, and protein decay—can potentially shape optimal intracellular abundance. Work studying the central dogma rates of fast growing human, mouse, yeast, and *E. coli* cells described governing principles including that these rates are balanced by optimizing the precision of control over abundance versus the production costs of mRNA and protein.^96^ When surveyed across genes Hausser et al. observed little gene-to-gene variation in the degradation rates of mRNA and protein. They then reasoned that abundance levels were primarily determined by the rates of transcription and translation. Further, they captured much of the observed variation in protein abundances with a model that balanced lethal stochastic variation at low expression levels with the cost of producing more mRNA. Interestingly, they concluded that control over transcription levels explained much of the observed control over protein abundance in the species they studied. Further work proposed a “robustness-load trade-off” model where the tolerance to variation in expression is balanced against the metabolic load of synthesizing the mRNA and protein molecules of a gene^86^ (Figure 2B). This model indicates that many genes are overexpressed relative to their critical protein abundance, with the degree of overexpression optimized to put the mean expression level high enough such that, even with noisy expression, most cells are above their fitness floor on average. Thus, expression is constrained by the balance of tolerable distance from the expression floor and the production costs of transcribing and translating the gene.

### Evolutionary Constraints on Relative Enzyme Abundance

In addition to constraints on the absolute abundance of an enzyme (i.e., how much is “enough” or “too much”), biochemical interactions can also drive constraints on *relative* abundance. That is, a pair of enzymes might be preferentially expressed in a certain ratio, or the abundance of one enzyme should exceed the other.^31^ For example, consider the network in Figure 2C. Decreasing the abundance of enzyme E2 could yield a fitness defect through different mechanisms: (1) depletion of E2′s product, (2) accumulation of E2′s substrate (if this leads to toxicity or off-target inhibition), or (3) accumulation of E1′s reaction product. The first case (product depletion) constrains the absolute expression level of E2, but cases 2 and 3 impose a constraint on the relative expression of E1 versus E2 through their shared substrate pool. Constraints on absolute vs relative protein abundance will give rise to distinct gene expression-fitness landscapes (Figure 2D).

A quantitative study using ribosome profiling showed that some metabolic enzymes are expressed in stoichiometric ratios conserved across domains of life,^79^ consistent with evolutionary constraints on the relative expression of these genes. Such coupling could be due to physical interactions such as metabolon subunits assembling for substrate channeling,^26^ or biochemical coupling through changes in metabolic flux as shown in Figure 2C.^14^ In some cases convergent evolution appears to have followed different mechanisms to arrive at similar stoichiometric ratios.^79^ Chromosomal proximity (synteny), shared transcription factors, and transcriptional units such as operons in bacteria can help maintain particular expression ratios.^97^ For example, yeast metabolic genes with toxic intermediate compounds are often found physically closer together in the genome (synteny) with increasing toxicity or reactivity of their associated intermediate compound(s).^98^ Studies perturbing gene expression or activity in a pairwise fashion^14,31,62,82^ have found that constraints on relative expression do exist, and the pathology of some inborn errors of metabolism, such as lysosomal storage diseases, are attributed to disrupted stoichiometry of components of metabolic pathways. Exogenous enzyme replacement or specialized diets were used for decades before the development of substrate reduction therapy, wherein small molecules inhibit enzymes producing intermediates that are toxic given the deficiency of their catabolizing enzyme. With increasing understanding of the constraints on pathway stoichiometry investigators are developing genetic approaches to substrate reduction therapy wherein the expression of genes proximal to a deficient enzyme are inhibited through RNA interference or similar approaches.^81^ Additional work is needed to determine the prevalence of these interactions. Observed sparsity of interactions may be due to true sparsity or may reflect that some interactions are only relevant in particular environmental conditions or nutrient limitations. We anticipate that future work quantifying the relationship between metabolic enzyme abundance and growth rate with tools like mismatch CRISPRi or orthogonal promoters will help map the constraints on both relative and absolute gene abundance.^14,99^

### Considering Cell Context in Synthetic Biology

Engineering of biosynthetic pathways begins by transferring natural or synthetic genes into a fast-growing and densely culturable “chassis” organism (e.g., a bacterium). This process has led to cost-effective production of the antimalarial artemisinic acid in yeast, biosynthesis of vinblastine precursors via a 30-step pathway, and carbon fixation in *E. coli* through a non-native Calvin cycle.^100–102^ However, just as cell context shapes the evolution of natural enzymes, it can restrict the space of viable engineered systems. Metabolic burden, the accumulation of toxic intermediates, inefficient gene expression due to incompatibilities in transcription and codon usage, and errors in protein folding, processing, or post-translational modifications can all reduce biosynthetic pathway yields or even render a system nonviable (Figure 3). For example, increased soil concentrations of the industrial solvent and environmental toxin dichloromethane (DCM) led to evolution of DCM catabolism in a *Methylobacterium extorquens* strain. A key enzyme in this process is a newly emerged DCM dehalogenase^103^ which enables utilization of DCM as a carbon source. However, the process of breaking down DCM releases chloride ions, disrupting physiological concentrations and driving toxicity. Careful analysis of changes in the genomes of organisms that have acquired the dehalogenase identified mutations which increased activity of a chloride exporter, allowing the dehalogenase to function in some genetic backgrounds (but not others).^104^ This knowledge of context can then be used to more effectively introduce DCM catabolism in other strains.

**Figure 3:**
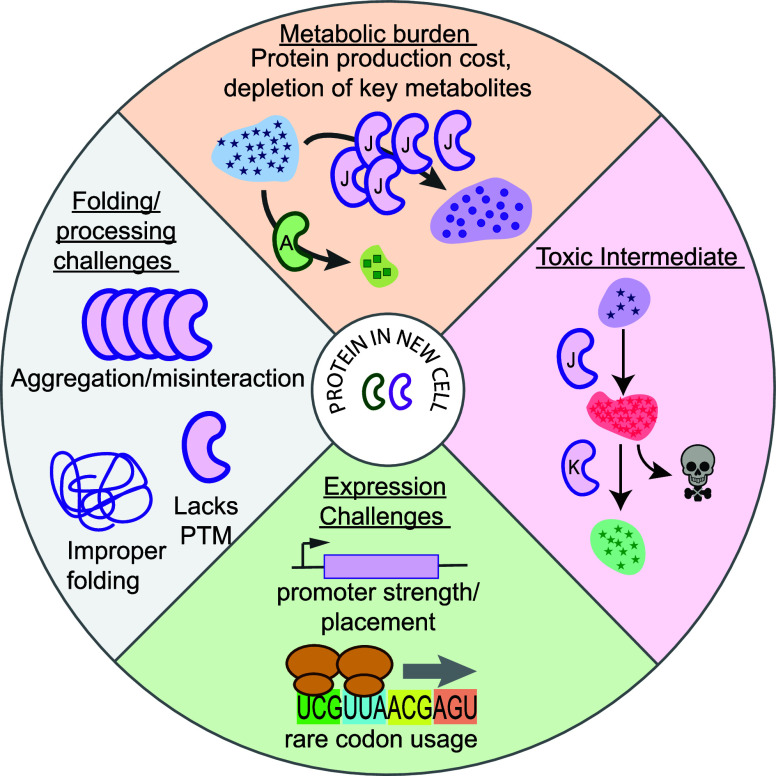
Challenges in engineering new metabolic functions. Metabolic burden. A heterologous enzyme (purple bean shape) can be expressed in a non-native cell context to synthesize a new biosynthetic product (purple circles). However, this may incur metabolic production costs associated with overexpression and deplete important metabolic intermediates (blue stars) necessary to produce key metabolites (green squares). Toxic Intermediates. Introduction of a new enzyme or biochemical pathway (purple bean shapes) can drive accumulation of toxic metabolic intermediates (red stars) that inhibit other enzymes and/or otherwise poison the cell. Expression challenges. Variation in codon usage (bottom schematic) and transcriptional machinery (top schematic) can limit the expression of heterologous genes. Incorrect folding or processing. In a new cell context, a heterologous protein (purple bean shape) may aggregate, incorrectly interact with other proteins, mis-fold, or lack post-translational modifications important to function.

Cell context can strongly impact heterologous protein expression due to variation in codon usage. Codon usage refers to the preference for specific synonymous codons in protein-coding genes, shaped by evolutionary and functional constraints.^105,106^ Natural variation in codon usage arises from mutational biases, translational efficiency, and genome composition, with highly expressed genes favoring codons that match abundant tRNAs to optimize translation.^107–112^ The fitness effects of synonymous codon substitution can be substantial; Agashe et al. found that encoding a key enzyme with only rare codons reduced fitness by 40%, while an allele encoded by frequent codons caused a >90% fitness loss due to variation in intracellular protein abundance.^113^ Incorrect folding or processing of heterologous proteins can significantly impair their functionality when expressed in non-native host environments. Drummond and Wilke showed that selection against misfolding-induced fitness costs slows the evolution of highly expressed proteins, where stability is favored to prevent toxic unfolded states.^112^

These challenges become compounded when engineering entire pathways where the coordinated expression of multiple enzymes can amplify metabolic strain. For instance, engineering the mevalonate (MVA) pathway for bisabolene production led to toxicity and reduced fitness due to unregulated expression of enzymes and misfolding. Similarly, in engineering *E. coli* for 1,3-butanediol (1,3-BDO) production, metabolic imbalance arose from the accumulation of toxic intermediates like pyruvate and 3-hydroxybutyric acid. In both cases, rational optimization of codon usage, enzyme expression, and metabolic flux were used to improve pathway stability and host viability.^114,115^

### Directed Evolution for Optimizing Engineered Systems

While rational optimization of pathways can prove powerful, it depends on prior mechanistic knowledge and may miss the contribution of nonintuitive or previously undiscovered factors. Directed evolution has thus become a common strategy to optimize the expression of key pathway genes and improve the functionality of specific pathways in microorganisms (Figure 4). Importantly these approaches—by necessity—consider cell context in the optimization process. Relying on native mutation rates and variation can be slow; many methods seek to elevate mutation rates and direct variation to genes of interest. Here we highlight three laboratory evolution methods that take distinct approaches to generating variation: Multiplex Automated Genome Engineering (MAGE), Orthogonal DNA Replication (OrthoRep), and Phage-assisted Continuous Evolution (PACE). MAGE uses recombination of DNA oligonucleotide (oligo) pools to introduce multiple, targeted genetic modifications into an organism’s genome (Figure 4A). This technique enables the generation of vast genetic diversity across a population of cells through the introduction of targeted mismatches, insertions, and deletions. As a result, MAGE facilitates screening a large set of defined variants for desired traits.^116–118^ MAGE can create targeted mutations throughout the genome, making it ideal for optimizing RBS or promoter variants in front of multiple genomically disperse genes. In contrast, OrthoRep offers a higher and sustained mutation rate within a specific genomic region that is independent of the host genome. In this method, originally pioneered in yeast, an Orthogonal DNA polymerase-plasmid (O–DNAP) system mutates target genes in the heterologous DNA approximately 100,000 times faster than the host genome, enabling rapid and continuous directed evolution.^119^ Because elevated mutation rate is restricted to a heterologous region of interest, this method reduces the likelihood of spurious mutations elsewhere in the genome. OrthoRep has been used to evolve drug-resistant malarial DHFRs and engineer *Thermotoga maritima* tryptophan synthase β-subunit (TmTrpB) variants with altered substrate profiles, demonstrating its potential for biomolecular evolution.^120,121^ Enhanced OrthoRep systems now achieve mutation rates exceeding 10^–4^ in yeast, and has recently been extended to two bacterial systems: *E. coli* and *Bacillus thuringiensis*.^122,123^ PACE is another technique that automates directed evolution by linking a target protein’s desired activity to the fitness of an infectious bacteriophage carrying the corresponding gene (Figure 4B). Unlike MAGE and OrthoRep, PACE confines mutations to a specific gene while leveraging the short generation times of bacteriophage to accelerate evolution. PACE operates in a continuous flow system, where host cells circulate through a “lagoon” and are infected by selection phages (SP) carrying a genetic library. Functional library members (Figure 4 middle path) produce pIII from accessory plasmid (AP), enabling progeny phages to infect new host cells, while nonfunctional members (Figure 4 lower path) cannot propagate. Mutagenesis is enhanced by a mutagenesis plasmid (MP), and mutations accumulate in the replicating phage population as host cells exit the lagoon faster than they replicate.^124^

**Figure 4:**
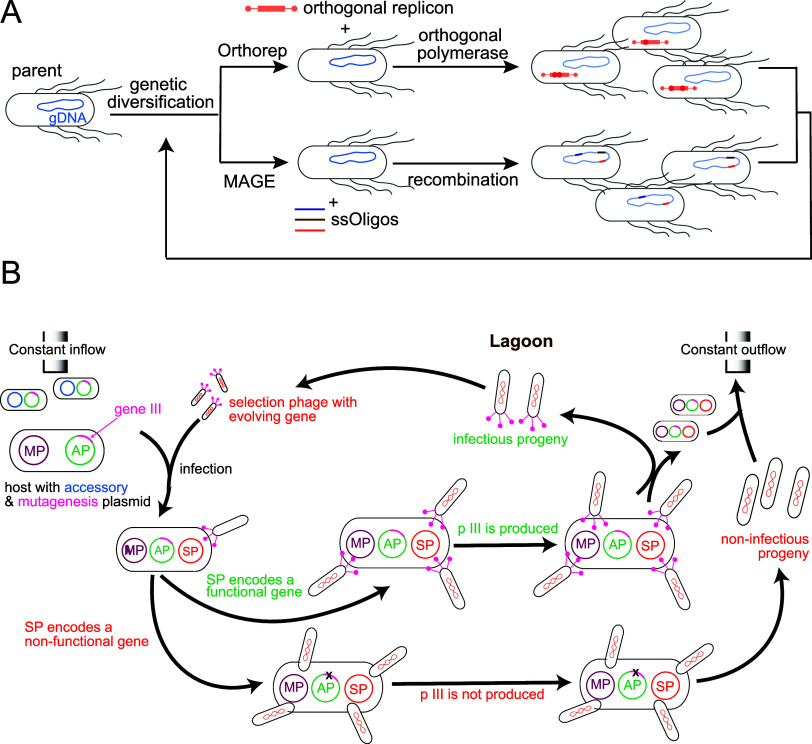
Laboratory evolution strategies for metabolic engineering. (A) Two strategies for accelerating genetic diversification in bacteria. In *E. coli* orthorep (EcORep, top fork of schematic) the genes targeted for mutagenesis and selection are placed on a linear dsDNA replicon (orange). This plasmid is replicated by an error-prone orthogonal DNA polymerase at far higher mutation rates (∼10^–7^) than the genome (∼10^–10^). In MAGE (bottom fork), competent *E. coli* are electroporated with a pool of single stranded oligos (purple, brown, orange lines). These fragments are then incorporated into the lagging strand of the host genome by a ssDNA binding protein derived from phage. (B) Phage assisted continuous evolution (PACE). In this strategy, the genes targeted for mutagenesis and selection are placed on the selection plasmid (SP, pink). Induction of the mutagenesis plasmid (MP, maroon) gives rise to continuous mutagenesis of SP. The accessory plasmid (AP, green) carries a gene encoding pIII, a protein necessary for phage infection. The system is initially designed so that production of pIII depends on the correct function of the SP genes. Successful pIII production gives rise to new infectious phage, which propagate the selection plasmid further. Figure adapted from Esvelt et al.^124^

MAGE, PACE, and OrthoRep can all be used to introduce genomic mutations in coding or regulatory regions. Alternatively, CRISPRi can be used to rapidly survey and optimize variation in gene expression levels without introducing mutations. For example, a tunable CRISPRi system was used to optimize flux distribution for violacein derivatives and lycopene production.^125^ Multiplexed CRISPRi in *E. coli* improved isoprenol yields by 4.5-fold through gene downregulation in the mevalonate and IPP bypass pathways.^126^ In another instance, an insulator-enhanced gRNA structure and off-switch Cas12m circuit ensure secure GMM management with low escape frequencies.^127^ The advantages of CRISPRi lie in its ability to screen large libraries, allowing for rapid exploration of expression variation across individual genes, pairs, or higher-order combinations. However, a limitation is its inherent instability, as “escapers” that no longer respond to CRISPRi can arise. This necessitates the implementation of alternative durable strategies, such as engineered promoters or ribosome binding sites (RBS), to encode stable optimal expression levels.^128^ Together, these strategies—MAGE, OrthoRep, PACE, and CRISPRi—represent powerful methods for optimizing gene expression and accelerating evolutionary processes in lab environments.

### Toward the Context-Aware Design of Synthetic Systems

Directed evolution is a powerful method for engineering proteins but can prove inefficient due to the complexities of developing a selection, the emergence of unintended responses to selective pressure, and the need for multiple selection rounds and parallel replicates. Moreover, the rugged nature of fitness landscapes makes it possible for systems to get stuck at a local optimum, leaving unreachable higher-fitness solutions undiscovered.^129^ In an ideal world, direct computational design of synthetic genes and pathways would bypass the need for iterative evolutionary optimization. Statistical and machine learning models of protein sequence hold substantial promise for this type of generative design.^53,130,131^ The central concept behind these approaches is to learn a probability density function describing the likelihood that a particular sequence belongs to a given protein family or functional class. Once the model is learned–typically from large and diverse alignments of natural sequences–it can be used to score the effect of mutations on fitness^132^ or design synthetic sequences by either sampling directly from the probability density function or a latent space.^34,133,134^ Machine learning models trained on sequence have designed new enzymes,^34,133^ fluorescent proteins,^135^ and much more. Yet they still struggle with efficiency, meaning that an appreciable fraction of the designed sequences does not fold or function as expected. Consequently, it is typical to synthesize libraries of hundreds or thousands of sequences and screen these through a high throughput functional assay to identify the fraction of successful designs.^131^

Two factors challenge our ability to design functional sequences and cell systems. The first is a lack of quantitative training data linking sequence to enzymatic activity and/or function. The development of AlphaFold hinged on the protein databank (PDB) as a rich source of 10^5^ high-quality structures.^136^ Because we lack an analogous resource for enzymatic function, many sequence-activity models are trained on natural homologous sequences under the assumption that these sequences share similar functional properties. This approach, sometimes called weak positive learning,^137^ is blind to potential evolutionary variation in specificity, activity, and other characteristics across homologues and still requires empirical measurements to validate its findings. Continued advances in high-throughput measurement of enzyme biochemical and biophysical properties will fuel new rounds of supervised learning; these measurements will be poised for maximal impact if they can be organized into a well-curated machine-readable database. The second challenge is that most computational design approaches generate sequences for individual protein targets one at a time without consideration of cell context. Given that even functional orthologs often fail to function properly in a new organism, it is then expected that a large fraction of context-agnostic sequence designs fail when assayed *in vivo*. We need new design processes that can better account for the role of cell context. Variational autoencoders (VAEs) offer one potential route forward. These methods encode information about protein sequence into a low dimensional latent space; decoding latent space vectors then yields new protein sequences. If the encoding is done properly, locality of sequences within the latent space reflects phylogenetic and functional relationships.^138^ Recent work indicates that sampling sequences from a local region of latent space is more likely to give rise to functional paralogs that bind a specific peptide and fulfill a particular cellular role.^134^ Thus, latent space representations may provide one strategy to identify sequences that function in a particular context. Another idea comes from large-scale genomic foundation models. Approaches like Evo, trained on a large collection of prokaryotic and archaeal genomes, are positioned to design stretches of DNA at a time.^139^ Evo has been used to design functional CRISPR systems composed of interacting with RNA and protein. By the nature of its whole-genome training, it presumably learns information about intermolecular interactions and cell context. A key question is how to define the relevant units of design. Should these be individual proteins, modules of biochemical pathways, or complete cellular processes? Moreover, because machine learning approaches are trained across many instantiations of a system (say learning from sequences sampled across species) they may miss idiosyncratic but important species-specific differences in activity and regulation. Tightly integrated design-build-test-learn loops are now needed to advance these and other generative sequence models. Together, we expect that improved training data and computational approaches for context-aware design will transform our ability to create new proteins and cell systems.^53,132,134,140–143^
